# Telomere clustering drives ALT

**DOI:** 10.18632/aging.102369

**Published:** 2019-10-13

**Authors:** Jaewon Min, Jerry W. Shay

**Affiliations:** 1 Department of Cell Biology, University of Texas Southwestern Medical Center, Dallas, TX 75390, USA

**Keywords:** telomeres, telomerase, BLM, RAD52, MiDAS, phase-separation, biomolecular condensates, PML bodies

Advanced human tumor cells universally need to acquire a telomere maintenance mechanism (TMM). While almost all carcinomas acquire a TMM by upregulating telomerase, there are some tumors that can divide indefinitely without activating telomerase. This is especially true in cancers of mesenchymal origin (such as sarcomas, endocrine tumors, glioblastoma, and some early childhood cancers). This telomerase-independent TMM has been termed Alternative Lengthening of Telomeres (ALT) [1]. ALT is a recombination-based telomere maintenance mechanism, however the underlying mechanisms by which the ALT pathway is initiated/mediated are still poorly understood. One of the hallmarks of ALT positive cancer specimens is the excessively clustered telomeres, shown as large bright telomere foci [2]. These telomere signals are clustered in promyelocytic leukemia (PML) bodies, known as ALT-associated PML Bodies (APBs) [3]. APBs contain telomeric DNA as well as many proteins involved in DNA replication, repair, and recombination processes. PML bodies are one of the nuclear membrane-less organelles which form by liquid-liquid phase separation organized by multivalent protein interactions between SUMO (Small Ubiquitin-like MOdifier) and SIM (SUMO-Interacting Motif) [4]. The underlying mechanisms by which telomere clustering in PML bodies are induced and how this is linked to the ALT pathway are not well understood.

**Figure 1:**
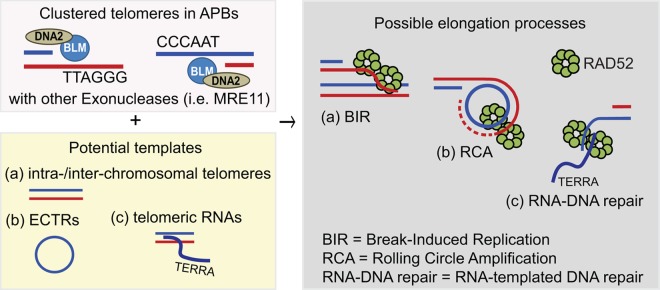
Schematic models for human ALT pathway.

We recently demonstrated a newly developed biophysical system that reconstitutes PML bodies from minimal components and generates telomere clustering, thus mimicking APBs in vivo [5]. By using polySUMO/polySIM condensates targeting telomeres, we demonstrated that the ALT mechanism in humans is triggered by excessive and persistent clustering of telomeres in PML bodies. Our observations help explain why ALT cancers display large bright telomere foci. Specifically, we demonstrated that these telomere foci are involved in telomere elongation processes. We further showed that the molecular mechanism of ALT is mediated by two stepwise functions of the BLM an RAD52 genes. ALT-like phenotypes are rapidly induced by introducing the APB-like condensates together with BLM overexpression in the presence of endogenous RAD52. We further determined that the underlying mechanism was associated with the helicase activity of BLM protein that is involved in 5’ to 3’ resection processes. In addition, the multimerization and DNA binding activity of the RAD52 protein, which has annealing activity, also participates in the ALT pathway. We demonstrated that the helicase activity of BLM is required for the initiation of telomere clustering and telomere synthesis through the generation of single-stranded telomeric DNAs via long-range resection processes. Consistent with our observations, it has been shown that the helicase activity of BLM is essential for the ALT activity in other organisms, i.e. *S. cerevisiae* and *U. maydis* [6]. We further demonstrated that RAD52 participates in ALT processes through its highly conserved N-Terminal Domain (NTD). The RAD52 NTD has the annealing activity between single-stranded DNA and potential templates, such as single stranded DNA, double stranded DNA, or RNA. Collectively, we proposed that RAD52 is involved in annealing the resected single-stranded telomeres to potential templates, i.e. chromosomal telomeres, extrachromosomal telomeric repeats, and telomeric non-coding RNA (TERRA), leading to telomere elongation through non-canonical recombination processes, such as break-induced replication, rolling circle amplification, and RNA-templated DNA repair (Figure 1).

Human ALT cancer cells exhibit severe DNA replication stresses at telomeres, which leads to SMC5/6-mediated SUMOylation at telomeres and telomere clustering in PML bodies (e.g. formation of APBs) [7]. Clustered telomeres in APBs are processed by long-rang resection reactions though BLM-DNA2 (helicase-endonuclease) and possibly other exonucleases (i.e. MRE11) [6,7] leading to the accumulation of single-stranded telomeric DNAs. Since most ALT cells do not have an intact G2/M checkpoint, ALT cells can bypass the checkpoint and undergo cell cycle progression with these exposed single-stranded telomeres [8]. Ultimately, these excessive amounts of single stranded telomeres clustered in APBs are repaired in late G2 or early M-phase by non-canonical recombination mediated by RAD52, whereas canonical recombination processes (i.e. homologous recombination) are suppressed by highly-enriched CDK activity in late G2/early M.

In conclusion, we generated artificially engineered APB-like condensates in vivo to study the ALT pathway [5]. Further refining our model system to more closely reflect spontaneous in vivo events (i.e. continuous cell proliferation and inducing telomere clustering in endogenous PML bodies) will provide further insights into the ALT pathway. Since APBs are the major site for telomeric synthesis during late G2/early M in ALT cells, a better understanding of the regulation of APB assembly/disassembly processes are likely to be crucial for the development of novel therapeutic strategies for ALT cancers in the future.
